# Enhanced Processing of Painful Emotions in Patients With Borderline Personality Disorder: A Functional Magnetic Resonance Imaging Study

**DOI:** 10.3389/fpsyt.2019.00357

**Published:** 2019-05-27

**Authors:** Vera Flasbeck, Björn Enzi, Martin Brüne

**Affiliations:** Division of Cognitive Neuropsychiatry and Psychiatric Preventive Medicine, Department of Psychiatry, Psychotherapy and Preventive Medicine, LWL University Hospital Bochum, Ruhr-University, Bochum, Germany

**Keywords:** empathy for pain, borderline personality disorder, functional magnetic resonance imaging, anterior insula, supramarginal gyrus

## Abstract

Previous research has demonstrated that patients with borderline personality disorder (BPD) are more sensitive to negative emotions and often show poor cognitive empathy, yet preserved or even superior emotional empathy. However, little is known about the neural correlates of empathy. Here, we examined empathy for pain in 20 patients with BPD and 19 healthy controls (HC) in a functional magnetic resonance imaging (fMRI) study, which comprised an empathy for pain paradigm showing facial emotions prior to hands exposed to painful stimuli. We found a selectively enhanced activation of the right supramarginal gyrus for painful hand pictures following painful facial expressions in BPD patients, and lower activation to nonpainful pictures following angry expressions. Patients with BPD showed less activation in the left supramarginal gyrus when viewing angry facial expressions compared to HC, independent of the pain condition. Moreover, we found differential activation of the left anterior insula, depending on the preceding facial expression exclusively in patients. The findings suggest that empathy for pain becomes selectively enhanced, depending on the emotional context information in patients with BPD. Another preliminary finding was an attenuated response to emotions in patients receiving psychotropic medication compared to unmedicated patients. These effects need to be replicated in larger samples. Together, increased activation during the observation of painful facial expressions seems to reflect emotional hypersensitivity in BPD.

## Introduction

Borderline personality disorder (BPD) is a severe psychiatric disorder that occurs in 1% to 6% of the general population (1). The disorder is characterized by fragile self-images, poor impulse control, emotional instability, and self-injurious behavior (2–4). Moreover, BPD is often accompanied by comorbid depression, posttraumatic stress disorder, eating disorders, and addiction (5).

With regard to social cognition, a growing body of literature suggests that patients with BPD experience difficulties in “mentalizing” (or “cognitive empathy”), which refers to the ability to reflect upon one’s own and others’ mental states in terms of intentions, beliefs, desires, or feelings (6, 7). In contrast, emotional empathy describes the representation of own and others’ emotions (8, 9) [for reviews, see Refs. (10, 11)]. Studies in BPD have shown that patients are unimpaired or even better than controls in emotional empathy, but perform more poorly in cognitive empathy tasks (12–14). Related to this, other research focusing on emotion recognition reported no general difference between BPD patients and healthy control (HC) participants, whereas other studies reported a hypersensitivity toward negative emotions and a tendency to ascribe negative emotions to even neutral facial expressions (15–20).

As regard the neuronal correlates of these processes in BPD, Dziobek and colleagues examined empathy by using the multifaceted empathy test (MET) in a neuroimaging paradigm. They described decreased activation of the left superior temporal sulcus and gyrus (STS/STG) in BPD associated with cognitive empathy and increased activation of the right middle insular cortex during emotional empathy (21). Moreover, consistent with the above mentioned behavioral studies, other work reported hyperactivation of the amygdala during the processing of social stimuli or emotional facial expressions implying threat (22–24).

A novel approach to the study of empathic processes has introduced tasks in which participants are asked to put themselves into the shoes of another individual who experiences somatic pain. Research has shown that psychologically healthy participants activate a neural network comprising brain regions that strongly overlap with those areas that are involved in first-person pain processing. The core areas of this “pain matrix” include the bilateral anterior insular cortex and medial/anterior cingulate cortex, and these regions are also activated when observing someone else in a painful situation (25, 26). However, empathy for pain and the activation of the pain matrix depend on several state- and trait-dependent factors that facilitate the strength of empathy, as for example the psychological stress level of the participant, the level of familiarity of the person exposed to the painful stimulus, and the level of habituation or suppression to the presented stimuli. For example, one study showed that clinicians may express attenuated empathic responses to pictures of syringes (10, 27–29). Thus, the magnitude of one’s empathy for pain seems to crucially depend on the social and individual context.

In a recent study, our group aimed to investigate whether the presentation of facial emotions prior to the observed bodily pain affected the activation of the pain matrix (30). Since the presentation of a facial expression prior to the pain stimulus creates a particular emotional context in which the pain occurs, the activation of the pain matrix may therefore vary due to the divergent processing of empathy for the contextual painful situation. Aside from an activation of the pain matrix, we found an increased response to pain in the left dorsolateral prefrontal cortex after the presentation of angry facial expressions, a region that is supposedly involved in top-down control of emotional responses to negatively valenced stimuli (30).

In the present study, we sought to examine the neuronal correlates of empathy for pain in patients with BPD. Specifically, we were interested in the question whether the presentation of facial expressions of emotions prior to the painful stimulus would alter the empathic response in participants with BPD. Aside from altered general emotional and empathic processing found in patients with BPD (31), previous research reported elevated thresholds for somatic pain in BPD (32–34). Two other studies reported that firsthand experience of somatic (heat) pain was also shown to be altered in patients with BPD, as shown by decreased activation in the amygdala and the anterior cingulate cortex during painful stimulation and increased response in the dorsolateral prefrontal cortex (35, 36). Thus, patients with BPD seem to show attenuated responses to the firsthand experience of pain, whereas there are no findings that point toward decreased empathy for pain processing. In our study, we hypothesized that patients with BPD would show a stronger activation of the “pain matrix” compared with controls, particularly when the painful image followed the observation of negative facial emotions. We were also interested in how activation patterns would correlate with subjective empathy ratings.

## Materials and Methods

### Participants

For the present study, 20 female in-patients diagnosed with BPD according to a structured interview [Strukturierte Klinische Interview für DSM-IV (SKID-II) for personality disorders] according to *Diagnostic and Statistical Manual of Mental Disorders, Fourth Edition* (DSM-IV) criteria [German version by Ref. (37)] were recruited from the LWL University Hospital in Bochum, Germany. Nineteen female HC participants were recruited *via* advertisement. HC were free of present or past psychiatric disorders as well as their first-degree relatives. Participants in both groups were Caucasians and needed to be free of neurological and severe physical illness (including pain-related illnesses). Patients with BPD were excluded if they suffered from psychotic or bipolar disorder or current substance abuse. Comorbid disorders and medication within the patients group are shown in **Table 1**. The authors assert that all procedures contributing to this work comply with the Helsinki Declaration of 1975, as revised in 2008. All participants gave their written informed consent after the nature of the procedures had been fully explained. The study was approved by the Ethics Committee of the Medical Faculty of the Ruhr-University Bochum.

**Table 1:** Comorbid disorders and medication of patients with borderline personality disorder (BPD) in absolute (*N*) and relative (%) quantity.

	*N*	%
Comorbid disorders of BPD patients		
Depressive episode	9	45
Posttraumatic stress disorder	4	20
Phobic disorder	1	5
Eating disorder	1	5
Cannabis misuse	3	15
Alcohol misuse	2	10
Other substance misuse	1	5
Medication		
Without regular medication	10	50
Antidepressant	6	30
Antipsychotic	2	10
Antidepressant and antipsychotic drugs	2	10

### Questionnaires and Behavioral Data

The Interpersonal Reactivity Index (IRI) (8) was used to assess self-reported empathic abilities that are suggested to reflect trait empathy. The questionnaire consist of 28 questions from which four subscales, namely, “perspective taking” (PT), “fantasy” (FS), “empathic concern” (EC), and “personal distress” (PD) are calculated. Here, it is important to note that high PT, FS, and EC reflect high trait empathy, whereas a high PD score indicates a high stress level that impairs empathy behavior. The Mehrfachwahl–Wortschatz–Intelligenztest Test (MWT-A) (38), a task that is similar to the “Spot-the Word-Task” developed by Baddeley (39), was used to examine verbal intelligence. For validation of the emotional pictures used in the functional magnetic resonance imaging (fMRI) paradigm, participants rated each picture regarding its emotional content (“angry,” “happy,” “neutral,” or “painful”) on a visual analog scale ranging between 10 and 90. As an additional question, participants were asked to indicate the gender of the depicted face. However, since we did not focus on a gender effect in the present study, the results obtained are not reported. The validation task took place after the fMRI scanning.

### Empathy for Pain Functional Magnetic Resonance Imaging Paradigm

**Figure 1:**
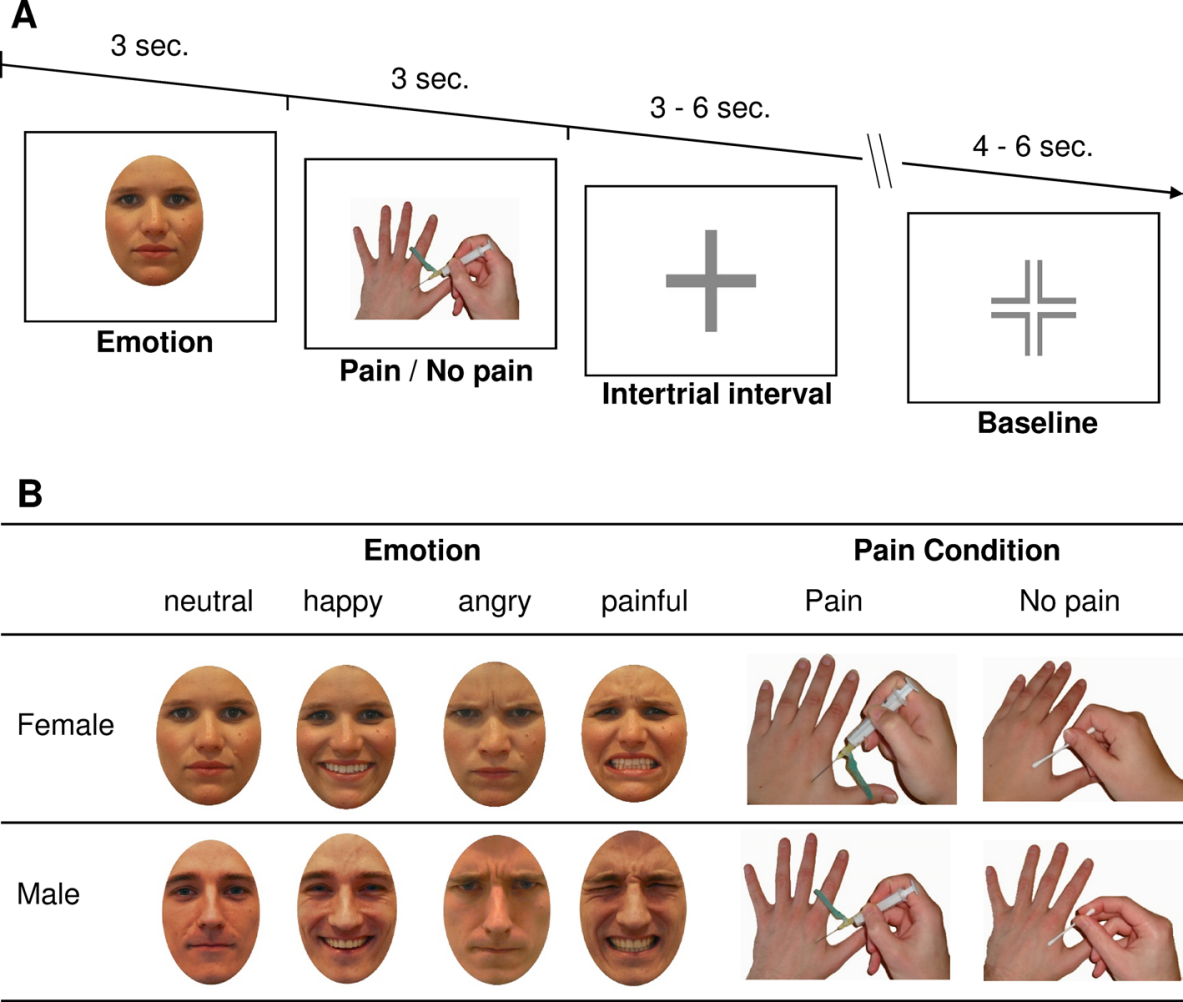
Description of the functional magnetic resonance imaging (fMRI) paradigm. Panel **(A)** shows the timing of one single trial of the empathy for pain paradigm, and Panel** (B)** shows exemplary pictures used in the paradigm showing the emotions “neutral,” “happy,” “angry,” and “painful” and the painful conditions “pain” and “no pain” for females and males, respectively.

The paradigm used in the present study was similar to an empathy-for-pain task developed by Lamm and colleagues (40) and was used in a similar way in our previous study (30). Briefly, a picture showing an emotional face (depicting an angry, happy, neutral, or painful facial expression) was presented to the participants for 3 s, followed directly by a hand exposed to a painful (needle penetrating the hand) or a nonpainful stimulus (Q-tip touching hand) for another 3 s. The trial ended with a jittered intertrial interval for 3–6 s and 10 occasional short breaks (4–6 s; **Figure 1**). The face and hand images were taken from Caucasian males or females (two different faces per gender); as a control condition, a black square was shown instead of faces preceding male hands. Participants were asked to empathize with the presented scenarios. In summary, each combination of facial emotion, gender, and pain condition was presented three times (control conditions six times), resulting in 60 trial presentations per run (30 painful and 30 nonpainful conditions), which together took approximately 11 min, with 4 runs leading to a total sum of 240 trials and 45 min. The inter-run-interval between run 1 and run 2 was in average 77.2 s (*SD* = 22.5 s; range 55.2–143.7 s), between run 2 and run 3 106.75 s (*SD* = 1; range 59.8–573.2 s), and between run 3 and run 4 82.7 s (*SD* = 19.1 s; range 61.9–137.8 s). After each run, participants were asked how well they could empathize by using a visual analog scale. More detailed, participants were asked to rate their success in feeling with the presented character (“empathy character”) and with the presented painful situation (“empathy pain”). Finally, they were asked to rate their current subjective well-being (“well-being”). The paradigm was presented using the “Presentation” software (Neurobehavioral Systems Inc., Albany, CA) *via* MRI-compatible liquid crystal display (LCD) goggles (Resonance Technology Inc., Los Angeles, CA).

### Functional Magnetic Resonance Imaging Acquisition and Data Analysis

The fMRI data were recorded using a 3-tesla whole-body MRI system (Philips Achieva 3.0T TX) and a 32-channel SENSE head coil. The MRI scan started with a high-resolution T1-weighted anatomical gradient echo scan (3D TFE: matrix 300 × 235 mm^2^, reconstructed to 320 × 320 mm^2^, field-of-view 240 × 188.8 × 192 mm^3^, in-plane resolution 0.8 × 0.8 mm^2^, slice thickness 0.8 mm, reconstructed to a final voxel size of 0.75 × 0.75 × 0.8 mm³). In total, 240 slices in transverse orientation were acquired (TR = 10 ms, TE = 4.6 ms, flip angle α = 8°, SENSE factor RRL = 2.5 and RFH = 2.0). Functional data during the empathy for pain paradigm were collected using T2*-weighted echo-planar imaging (EPI) sequences. Thirty-two slices were acquired in interleaved order parallel to the bicommissural plane. To obtain blood-oxygen level-dependent (BOLD) contrasts, we used a sensitivity encoded single-shot echo-planar imaging protocol (SENSE-sshEPI: number of slices 32, matrix 80 × 80 mm^2^, reconstructed to 112 × 112 mm^2^, field-of-view 220 × 220 mm^2^, in-plane resolution 2.75 × 2.75 mm^2^, slice thickness 3 mm with 1 mm gap, reconstructed to a final voxel size of 1.96 × 1.96 × 3 mm^3^, TR = 2,000 ms, TE = 30 ms, flip angle α = 90°, SENSE factor RAP = 2.0). The EPI sequence started with five scans that were discarded due to saturation effects. Every run contained 335 volumes and takes approximately 11 min. In total, participants completed four scanning runs.

The collected fMRI data were preprocessed and analyzed statistically using SPM12 (Wellcome Trust Center for Neuroimaging, Institute of Neurology, University College London, UK; http://www.fil.ion.ucl.ac.uk) and MATLAB 7.11 (The MathWorks Inc, Natick, MA). Preprocessing of the data implies slice timing correction, realignment, coregistration, and normalization with a T1 template provided by Statistical Parametric Mapping (SPM). The images were smoothed with an isotropic 8-mm full-width half-maximum Gaussian kernel, and the final voxel size of resampled images was 2 × 2 × 2 mm³. We applied a high-pass filter (cutoff, 100 s) to eliminate low-frequency signal drifts. Based on our previous study (30), we focused the analyses on the phase of pain/no pain perception according to the preceding emotional facial expression [e.g., (angry face+pain), (happy face+no pain), etc]. Thus, at the single subject level, regressors were combinations of pain condition, emotion, and gender. The realignment parameters were entered as regressors of no interest in the design matrix. A statistical model for each participant was calculated by convolving a hemodynamic response function with the abovementioned design (41). Subsequent statistical analysis followed the general model approach (42). As proposed by Poldrack and colleagues (43), and already used in social cognition research (44), we focused our analysis on hypothesis-driven regions of interest (ROIs) known to be involved in empathy for pain. To this end, we designed a mask containing the ROIs by using the WFU PickAtlas (45). All ROIs were chosen in accordance with a recent meta-analysis by Lamm and colleagues (26). The following ROIs were included: the anterior bilateral insula, the left medial cingulate cortex, the bilateral supramarginal gyri, the bilateral pallidum, the bilateral inferior temporal gyri, the bilateral amygdala, the left precentral gyrus, the right frontal inferior gyrus (pars opercularis), and the left thalamus. To visualize the brain areas involved in pain processing, the so-called pain matrix, we examined the T-contrast “effect of pain,” that is, [pain > no pain] collapsed over all emotions and gender using the “full factorial” option in SPM. This options shows activations with *p*[uncorrected] *<*0.001 for an extent k > 10 voxel. To deal with the still existing multiple testing problem and in accordance with the developers of the WFU PickAtlas software, peak voxel FWE correction was applied and only activation surviving a threshold of *p*[FWE] < 0.05 was considered significant. All activations were labeled according to the anatomical automatic labeling (AAL) atlas (46) implemented in the WFU PickAtlas (45). Afterward, percent signal changes from the abovementioned ROIs that showed activations were extracted using the “MarsBar” toolbox (http://marsbar.sourceforge.net/) for SPM12 (47). In a more fine-grained analysis, percent signal changes were further analyzed regarding the facial expression using SPSS 25.0. By using this localizer-based approach, we aimed to avoid the problem of “double dipping” (48). In the **Supplemental Material**, we show additional alternative analyses for further confirmation of the findings.

### Statistical Analysis

Further statistical analyses were performed using “IBM SPSS Statistics for Windows,” version 25 (IBM Corp., Armonk, NY). The differences between groups in questionnaires were examined by independent sample *t*-tests. For comparison of the frequency of distribution regarding the participant’s handedness, we calculated Fisher’s exact test (two-sided). Behavioral data were investigated using mixed-model ANOVA with the factors presented “facial expression” (i.e., angry, happy, neutral, or painful pictures) and identification of the emotion (angry, happy, neutral, or painful), that is, the response of participants and the between subject factor group (BPD, control). One-sample *t*-tests were used to assess whether category ratings differed significantly from the value 50, which was the center of the visual scale and therefore 50 indicates inconclusiveness in attribution of picture descriptions. To investigate whether habituation occurred, we calculated mixed-model ANOVAs with the within-subject factor “run” (runs 1–4) and the between-subject factor group (BPD, control). The ANOVA was calculated for each question separately (“empathy character,” “empathy pain,” and “well-being”) and for the reaction time, which was defined as the initial reaction on the first question at the end of each block.

The fMRI data were analyzed by mixed-model ANOVAs with the factors, pain condition (pain/no pain), facial emotion (angry, happy, neutral, and painful, no emotion), and group (BPD/HC), for each region separately. We calculated an additional mixed-model ANOVA including only patients with BPD and the within-subject factors pain condition (pain/no pain) and facial emotion (angry, happy, neutral, and painful, no emotion) and the between-subject factor medication (patients with BPD receiving medication and patients free of medication), for each region separately. Dependent and independent *t*-tests were used for *post hoc* comparisons. All ANOVA results reported were Greenhouse–Geisser corrected. According to the work of Costantini et al., we calculated correlations between IRI scores and brain activity during painful conditions only for the supramarginal gyri (49). In detail, we calculated Pearson correlation coefficients for each IRI subscale and activation during “pain” conditions pooled for emotional faces. We further corrected for multiple testing with results considered significant only if *p* < 0.05/4 = 0.0125.

## Results

### Participant Characteristics

We found significant differences between groups for IRI PT and PD scores (see **Table 2**), but not for age and IQ and handedness.

**Table 2:** Participant characteristics and results of comparisons of Interpersonal Reactivity Index (IRI) results (M = mean and range, SD = standard deviation) between patients with BPD and healthy controls (HC). *T*-test statistics (*t*, *p*, and Cohen’s *d*) are reported. For the comparison of handedness, Fisher’s exact test was calculated and the *p*-value (two-sided exact test value) is reported.

	BPD		HC		Test statistics		
	*M* (range)	*SD*	*M* (range)	*SD*	*t*	*p*	Cohen’s *d*
Age	26.1 (18–39)	6.4	23.4 (18–48)	6.2	1.28	0.207	0.41
IQ	106.6	19.6	108.6	15.5	−0.36	0.722	0.11
Handedness (right/left/ambidextrous)	17/2/1		18/1/0			1.000	
IRI perspective taking	15.2	5.6	18.7	4.5	−2.12	0.041	0.69
IRI fantasy	20.4	5.6	19.1	4.5	0.77	0.446	0.25
IRI empathic concern	20.3	4.1	18.9	4.6	0.92	0.362	0.30
IRI personal distress	21.0	5.4	10.6	3.8	6.80	0.001	2.25

### Behavioral Data

The mixed-model ANOVA with the factors “facial expression” and “identification” and group revealed a significant main effect of facial expression (*F*(2.3, 73.7) = 9.11, *p* < 0.001) and identification (*F*(2.6, 88.1) = 15.81, *p* < 0.001) and the interaction facial expression–identification (*F*(3.3, 106.3) = 391.40, *p* < 0.001), indicating selective rating depending on facial expression and identification. Importantly, no main effect or interaction with group appeared, showing that both patients and controls recognized the emotional content equally well. In addition, participants recognized the emotions correctly as indicated by significantly higher ratings than the “inconclusive value” of 50 (angry expressions rated as angry: *t*(33) = 17.49, *p* < 0.001; happy expressions rated as happy *t*(33) = 37.65, *p* < 0.001; neutral facial expressions rated as neutral *t*(33) = 7.05, *p* < 0.001; painful facial expressions rated as painful *t*(33) = 19.78, *p* < 0.001). All other comparisons (e.g., angry faces described as neutral) reached significance with values lower than 50, which stands for rebuttal of the suggested emotion category. In other words, participants did not mistake any emotion for another. For the behavioral results, ratings of one patient and four controls are missing due to timing/technical problems (BPD, *n* = 19; HC, *n* = 15).

The analyses of behavioral data during the fMRI task aimed to check whether participants habituated to the task over the four runs and whether a difference in subjective empathy occurred between patients with BPD and controls. First, the mixed-model ANOVA with the factors “run’’ and “group” did not show any main effects or interactions (“empathy character”: main effect “run”: *F*(2.0, 59.2) = 0.22, *p* = 0.804; interaction run–group *F*(1.97) = 1.92, *p* = 0.156; “empathy pain”: main effect “run”: *F*(2.2, 67.4) = 0.88, *p* = 0.432; interaction run–group *F*(2.25) = 1.73, *p* = 0.181; and “well-being”: main effect “run”: *F*(2.5, 75.0) = 1.52, *p* = 0.221; interaction run–group *F*(2.50) = 1.25, *p* = 0.295). Thus, rating did not change over time and did not differ between groups.

We further compared the reaction time of the initial reaction on the first response screen to check whether participants attended constantly to the task. Here, the ANOVA did not show any main effects or interactions (main effect “run”: *F*(2.1, 64.3) = 1.65, *p* = 0.199; interaction run–group *F*(2.14) = 0.15, *p* = 0.875), which indicates that participants attended constantly to the task with no difference between groups.

### Functional Imaging Data

**Figure 2:**
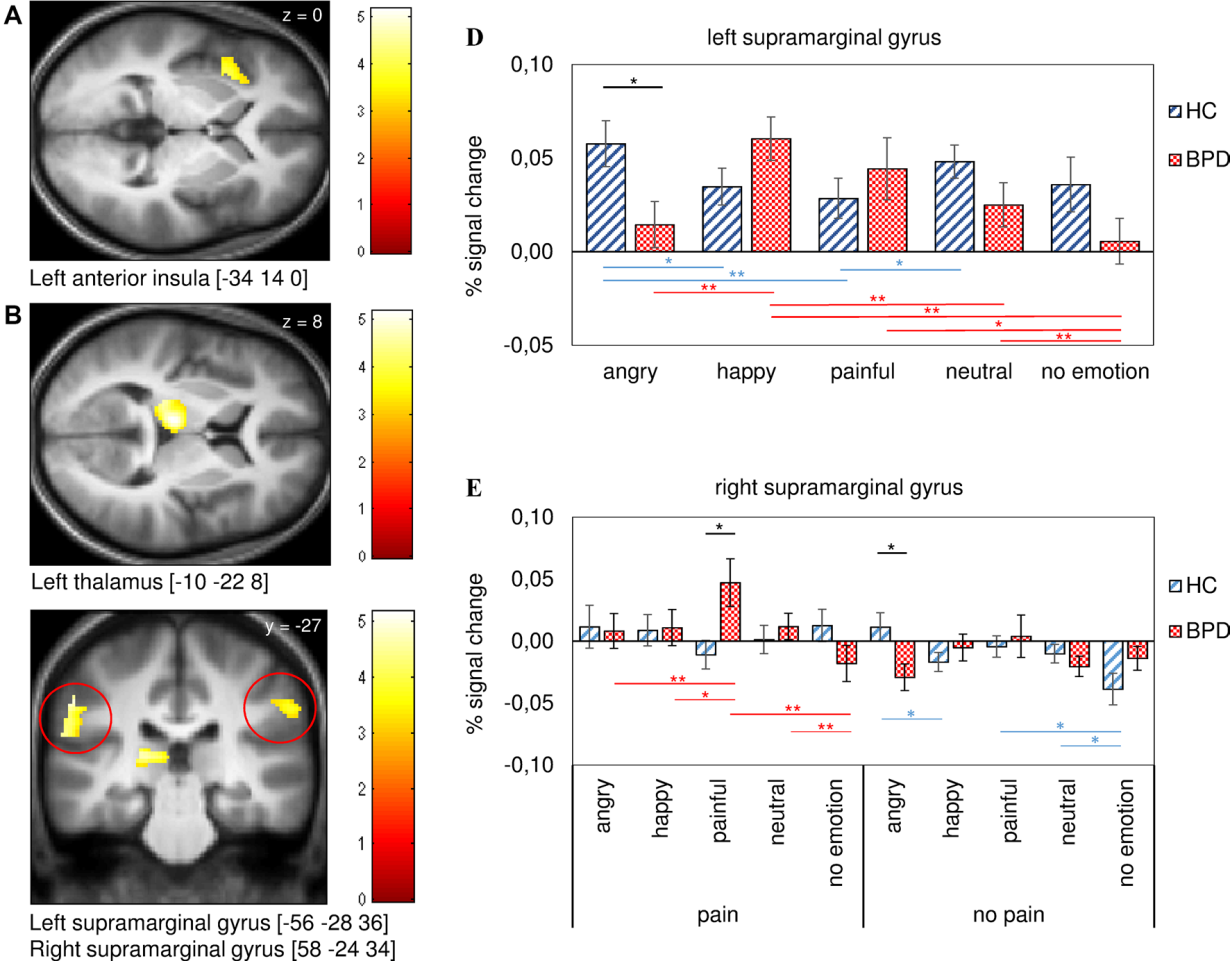
Activation and signal change in percent for regions of interest (ROIs) derived from the contrast (positive effect of pain) for patients with BPD and healthy controls (HC). Regions shown are the left anterior insula **(A)**, the left thalamus **(B)**, and the left and right supramarginal gyri **(C)**. The statistical parametric maps show a threshold of *p* [uncorr]. < 0.001 for *k* > 10. The diagram in Panel **(D)** shows differences between groups for the facial expressions in the left supramarginal gyrus and the diagram in Panel E contains pain and no pain conditions with preceding facial emotions in the right supramarginal gyrus. Note that differences between groups are marked in black, differences within the BPD group are marked in red, and within the control group in blue. We decided to exclude differences between condition (e.g., [angry face+no pain] vs. [angry face+pain] from diagrams to reduce confusing labeling. Error bars represent standard error of the mean (SEM) and *p < 0.05, **p < 0.010, ***p < 0.001.

Investigation of the contrast “effect of pain versus no pain” showed activation of the left thalamus, the left anterior insula, and bilateral supramarginal gyri (**Table 3**). For these regions, statistical analysis by mixed-model ANOVA were performed with the factors condition (pain, no pain), facial emotion (angry, painful, happy, neutral, no emotion) and group (BPD and HC). Accordingly, we found a significant main effect of “condition” for the left insula (*F*(1, 37) = 5.92, *p* = 0.020), the left thalamus (*F*(1, 37) = 7.53, *p* = 0.030), left supramarginal gyrus (*F*(1, 37) = 29.08, *p* < 0.001), and right supramarginal gyrus (*F*(1, 37) = 10.84, *p* = 0.002) (**Figure 2A–C**). Further *post hoc* comparisons showed that activation during “pain” trials differed in all regions from activation during “no pain” trials (left insula *t*(38) = 2.08, *p* = 0.045, left thalamus *t*(38) = 2.909, *p* = 0.006, left supramarginal gyrus *t*(38) = 4.86, *p* < 0.001, and right supramarginal gyrus *t*(38) = 3.93, *p* < 0.001).

**Table 3:** Activated brain regions for the contrast [positive effect of pain] collapsed over gender and emotions.

Region name	Hemisphere (left/right)	Coordinates (MNI)	*t* value	*z* value	*p_FWE-corr_*
*T* Contrast: [pain > no pain] collapsed over all emotions and groups					
Anterior insula	Left	−34 14 0	3.90	3.78	0.039
Thalamus	Left	−10 −22 8	5.18	5.08	<0.001
Supramarginal gyrus	Left	−56 −28 36	4.82	4.75	0.001
Supramarginal gyrus	Right	58 −24 34	3.98	3.94	0.034

Initial threshold p[uncorr]. < 0.001 for k > 10 voxel.

FWE correction with p < 0.05 on voxel level.

MNI, Montreal Neuroimaging Institute.

Moreover, we discovered a “condition–emotion–group” interaction for the left insula (*F*(3.18) = 3.01, *p* = 0.030) and the right supramarginal gyrus (*F*(3.63) = 4.71, *p* = 0.002). Independent *t*-test showed differences between groups for responses to painful pictures following the presentation of painful faces [painful face+pain] (*t*(37) = 2.56, *p* = 0.015) and to nonpainful pictures after angry faces [angry face+no pain] (*t*(37) = −2.60, *p* = 0.013) for the right supramarginal gyrus (**Figure 2E**). We further observed differences within groups for painful emotional conditions compared with the same emotional but nonpainful condition (see **Supplemental Table S2**).

In the BPD group, we found differences for [angry face+pain] versus [neutral face+pain] in the left insula *t*(19) = −2.16, *p* = 0.044), [painful face+pain] versus [no emotion+pain] (left insula *t*(19) = 3.05, *p* = 0.007; right supramarginal gyrus *t*(19) = 3.29, *p* = 0.004), and for [neutral face+pain] versus [no emotion+pain] (left insula *t*(19) = 4.04, *p* = 0.001; right supramarginal gyrus *t*(19) = 2.96, *p* = 0.008). Further differences were found in the right supramarginal gyrus for [angry face+pain] versus [painful face+pain] (*t*(19) = −3.22, *p* = 0.005) and for [painful face+pain] versus [happy face+pain] (*t*(19) = 2.33, *p* = 0.031) **Figure 2E** shows comparisons in the right supramarginal gyrus; comparisons in the anterior insula are not shown.

Significant differences, though to a lesser degree, were also found in the control group, showing differences only within the nonpainful conditions for [neutral face+no pain] versus [no emotion+no pain] (*t*(18) = 2.18, *p* = 0.043), [angry face+no pain] versus [happy face+no pain] (*t*(18) = 2.57, *p* = 0.019), and [painful face+no pain] versus [no emotion+no pain] (*t*(18) = 2.38, *p* = 0.028) in the right supramarginal gyrus.

Finally, the ANOVA revealed a significant interaction of emotion-with group for the left (*F*(3.24) = 6.07, *p* < 0.001) and the right supramarginal gyrus (*F*(2.89) = 3.13, *p* = 0.030). Here, independent *t*-tests between groups showed a significant difference for angry faces for the left supramarginal gyrus (*t*(37) = −2.48, *p* = 0.018; see **Figure 2D**). Further *post hoc* comparisons within groups showed differences in activation between the emotions. These differences were apparent in both groups but showed a tendency toward more significant differences between emotions in patients with BPD (see **Figure 2D** and **Supplemental Table S1**).

**Figure 3:**
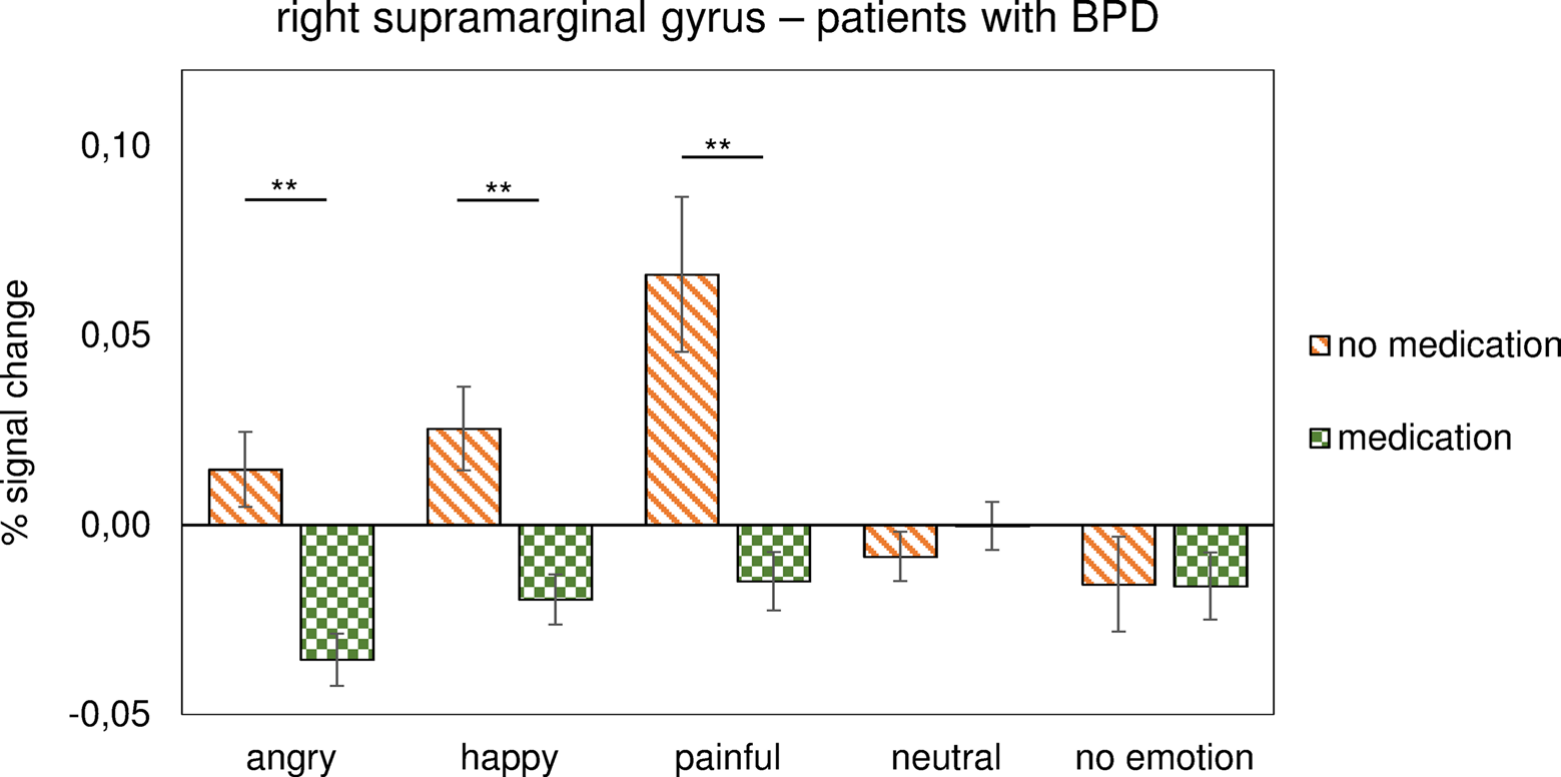
Activation during emotional face processing in percent signal change for the right supramarginal gyrus in patients with BPD. The diagram compares patients under medication with unmedicated patients. Error bars represent SEM and *p < 0.05, **p < 0.010, ***p < 0.001.

An additional explorative mixed-model ANOVA with the within subject factors pain condition (pain/no pain), facial emotion (angry, happy, neutral, and painful, no emotion), and medication (BPD with medication/BPD without medication) revealed a significant interaction of emotion–medication for the right supramarginal gyrus (*F*(2.13 = 5.90, *p* = 0.005). Independent *t*-test of medicated vs. unmedicated patients with BPD further revealed a difference in response to angry, happy, and painful faces (angry *t*(18) = 4.16, *p* = 0.001; happy *t*(18) = 2.47, *p* = 0.024; painful *t*(18) = 2.63, *p* = 0.017; **Figure 3**).

These results indicate that patients with BPD showed lower activation regarding angry, painful, and happy facial expressions under medication when compared with unmedicated patients. The other main effects and interactions detected by the ANOVA are listed in **Supplementary Table S3**.

Regarding subjective reports, we found a significant correlation between the IRI subscale “perspective taking” and brain activation during “pain” trials (*r* *_s_* (37) = 0.409, *p* = 0.012).

## Discussion

The present study aimed to investigate the neural correlates of empathy for pain combined with emotional facial expressions in patients with BPD using fMRI. Behaviorally, patients scored significantly higher on the alexithymia questionnaire and reported higher PD and lower PT on the IRI questionnaire. However, ratings of facial emotions used in the fMRI paradigm did not differ between groups, indicating that both patients and HC recognized the emotional expressions equally well. In our fMRI analysis, we focused on the contrast “effect pain” [pain > no pain] for hypothesis-driven ROIs derived from previous neuroimaging work on empathy for pain (26). Most importantly, we found significant interactions of condition–emotion–group and emotion–group for the left and right supramarginal gyri. *Post hoc* test revealed that patients showed significantly higher activations to painful pictures following the presentation of painful faces in the right supramarginal gyrus. Lower activation was discovered for nonpainful pictures following angry faces in the right supramarginal gyrus. In addition, patients with BPD generally showed lower activation in the left supramarginal gyrus when viewing angry facial expressions compared to HC.

This finding is consistent with Van der Heiden et al.’s study who found enhanced activation in the left supramarginal gyrus in an empathy-for-pain task in which psychologically healthy participants were asked to adopt the “Self”-perspective compared to the “Other”-perspective (35). Moreover, Costantini and colleagues reported activations in the left and right supramarginal gyri during an empathy-for-pain task. They further found significant correlations between these activations and the PT subscale of the IRI (49), which was replicated in our present study for the left supramarginal gyrus. Our findings are also in line with those of previous studies in healthy participants, suggesting that increased activation within the insula may correspond to the subjective experience of negative emotions, including disgust, fear, and anger (50–52), and enhanced activity in the same region in patients with BPD (53–55). Consistent with this idea, comparisons between conditions and emotions revealed several significant differences within the patient group in terms of insula activation, but no such differences in the control group. These findings may reflect differences in emotion processing, with more pronounced activations in response to painful faces and an attenuated response to angry faces in BPD relative to controls. Another possible reason for the weaker response to angry faces could be that anger usually does not fit into the context of pain (especially nonpainful conditions) and thus does not lead to activation of the empathy for pain network.

In the present study, we did not observe between-group differences in general processing of empathy for pain. Previous research reported increased thresholds for somatic pain in BPD and altered neuronal processing during painful stimulation (32–34, 36, 53). However, reduced sensitivity to physical pain in BPD does not automatically translate into attenuated empathetic responsivity. Instead, we contend that patients with BPD are overly responsive to another’s bodily pain, specifically, when the painful situation is cast in a social cognitive context, such as facial emotions suggestive of how the person experiencing the pain responds to it emotionally. Put another way, we do not conclude from our data that patients with BPD are impaired in their ability to empathize with another person exposed to somatic pain—rather, their “smoke detector” is more sensitive to potentially threatening or otherwise aversive situations (56).

Another important result that should be considered as preliminary is the impact of medication in empathy for pain. In the present study, medication seems to decrease the patients’ neural response to emotional faces (angry, happy, and painful faces) in the right supramarginal gyrus. Due to the small sample size and the uncontrolled nature of the comparisons regarding medication, we caution that these results need to be replicated in larger samples.

The current study has several limitations. First, since we recruited only female participants, the results are not generalizable for both genders. Second, we did not include a clinical control group, so, it is unclear whether our findings are specific to BPD. Third, patients with BPD were in an inpatient psychotherapy setting, and we were unable to control for potential therapeutic effects. Fourth, the comorbidity pattern, especially the presence of comorbid depression or posttraumatic stress disorder, may have influenced our findings. Fifth, since we performed the diagnostic interview only with patients, the control group was less carefully examined for potential psychological problems. Finally, the small and heterogeneous sample (e.g., with regard to eligibility criteria or medication as mentioned above) may have lowered statistical power. In summary, our study suggests that patients with BPD display heightened sensitivity to another’s somatic pain, especially when the observed pain stimulus is preceded by a painful facial expression. Future research may utilize these insights including the study of longitudinal effects of psychotherapeutic treatment on empathy for physical pain.

## Ethics Statement

The authors assert that all procedures contributing to this work comply with the Helsinki Declaration of 1975, as revised in 2008. All participants gave their written informed consent after the nature of the procedures had been fully explained. The study was approved by the Ethics Committee of the Medical Faculty of the Ruhr-University Bochum.

## Author Contributions

VF: Study design, fMRI scanning, data analysis, manuscript writing. BE: Study design, fMRI scanning, data analysis, manuscript writing. MB: Study design, manuscript writing, and editing. All authors have approved the final article.

## Funding

This research did not receive any specific grant from funding agencies in the public, commercial, or not-for-profit sectors.

## Conflict of Interest Statement

The authors declare that the research was conducted in the absence of any commercial or financial relationships that could be construed as a potential conflict of interest.

## References

[1] Sansone RA Sansone LA Personality disorders: a nation-based perspective on prevalence. Innov Clin Neurosci (2011) 8(4):13–8 https://www.ncbi.nlm.nih.gov/pubmed/21637629 PMC3105841 21637629

[2] Gunderson JG Disturbed relationships as a phenotype for borderline personality disorder. Am J Psychiatry (2007) 164(11):1637–40. 10.1176/appi.ajp.2007.07071125 17974925

[3] Gunderson JG Links PS Borderline personality disorder: a clinical guide. Washington DC: American Psychiatric Pub (2009).

[4] American Psychiatric Association Diagnostic and statistical manual of mental disorders: DSM-5 -5. Washington, DC: American Psychiatric Pub (2013). 10.1176/appi.books.9780890425596

[5] Pagura J Stein MB Bolton JM Cox BJ Grant B Sareen J Comorbidity of borderline personality disorder and posttraumatic stress disorder in the US population. J Psychiatr Res (2010) 44(16):1190–8. 10.1016/j.jpsychires.2010.04.016 PMC4209725 20537660

[6] Fonagy P Attachment and borderline personality disorder. J Am Psychoanal Assoc (2000) 48:1129–46 [discussion 1175–1187]. 10.1177/00030651000480040701 11212185

[7] Fonagy P Luyten PA A developmental, mentalization-based approach to the understanding and treatment of borderline personality disorder. Dev Psychopathol (2009) 21:1355–81. 10.1017/S0954579409990198 19825272

[8] Davis MH Measuring individual differences in empathy: evidence for a multidimensional approach. J Pers Soc Psychol (1983) 44(1):113–26. 10.1037/0022-3514.44.1.113

[9] Davis MH Hull JG Young RD Warren GG Emotional reactions to dramatic film stimuli: the influence of cognitive and emotional empathy. J Pers Soc Psychol (1987) 52:126–33. 10.1037/0022-3514.52.1.126 3820067

[10] Gonzalez-Liencres C Shamay-Tsoory SG Brüne M Towards a neuroscience of empathy: ontogeny, phylogeny,brain mechanisms, context and psychopathology. Neurosci Biobehav Rev (2013) 37:1537–48. 10.1016/j.neubiorev.2013.05.001 23680700

[11] Singer T The neuronal basis and ontogeny of empathy and mind reading: review of literature and implications for future research. Neurosci Biobehav Rev (2006) 30:855–63. 10.1016/j.neubiorev.2006.06.011 16904182

[12] Harari H Shamay-Tsoory SG Ravid M Levkovitz Y Double dissociation between cognitive and affective empathy in borderline personality disorder. Psychiatry Res (2010) 175(3):277–9. 10.1016/j.psychres.2009.03.002 20045198

[13] Preißler S Dziobek I Ritter K Heekeren HR Roepke S Social cognition in borderline personality disorder: evidence for disturbed recognition of the emotions, thoughts, and intentions of others. Front Behav Neurosci (2010) 4:182. 10.3389/fnbeh.2010.00182 21151817 PMC2999836

[14] Niedtfeld I Experimental investigation of cognitive and affective empathy in borderline personality disorder: effects of ambiguity in multimodal social information processing. Psychiatry Res (2017) 253:58–63. 10.1016/j.psychres.2017.03.037 28351003

[15] Wagner AW Linehan MM Facial expression recognition ability among women with borderline personality disorder: implications for emotion regulation? J Pers Disord (1999) 13(4):329–44. 10.1521/pedi.1999.13.4.329 10633314

[16] Domes G Czieschnek D Weidler F Berger C Fast K Herpertz SC Recognition of facial affect in borderline personality disorder. J Pers Disord (2008) 22(2):135–47. 10.1521/pedi.2008.22.2.135 18419234

[17] Domes G Schulze L Herpertz SC Emotion recognition in borderline personality disorder—a review of the literature. J Pers Disord (2009) 23(1):6–19. 10.1521/pedi.2009.23.1.6 19267658

[18] Fertuck EA Jekal A Song I Wyman B Morris MC Wilson ST Enhanced ‘Reading the Mind in the Eyes’ in borderline personality disorder compared to healthy controls. Psychol Med (2009) 39(12):1979–88. 10.1017/S003329170900600X PMC3427787 19460187

[19] Daros AR Zakzanis KK Ruocco AC Facial emotion recognition in borderline personality disorder. Psychol Med (2013) 43(09):1953–63. 10.1017/S0033291712002607 23149223

[20] Wingenfeld K Kuehl LK Janke K Hinkelmann K Dziobek I Fleischer Enhanced emotional empathy after mineralocorticoid receptor stimulation in women with borderline personality disorder and healthy women. Neuropsychopharmacology (2014) 39(8):1799. 10.1038/npp.2014.36 24535100 PMC4059897

[21] Dziobek I Preißler S Grozdanovic Z Heuser I Heekeren HR Roepke S Neuronal correlates of altered empathy and social cognition in borderline personality disorder. Neuroimage (2011) 57(2):539–48. 10.1016/j.neuroimage.2011.05.005 21586330

[22] Herpertz SC Dietrich TM Wenning B Krings T Erberich SG Willmes K Evidence of abnormal amygdala functioning in borderline personality disorder: a functional MRI study. Biol Psychiatry (2001) 50(4):292–8. 10.1016/S0006-3223(01)01075-7 11522264

[23] Donegan NH Sanislow CA Blumberg HP Fulbright RK Lacadie C Skudlarski P Amygdala hyperreactivity in borderline personality disorder: implications for emotional dysregulation. Biol Psychiatry (2003) 54(11):1284–93. 10.1016/S0006-3223(03)00636-X 14643096

[24] Koenigsberg HW Siever J Lee H Pizzarello S New AS Goodman M Neural correlates of emotion processing in borderline personality disorder. Psychiatry Research: Neuroimaging (2009) 172(3):192–9. 10.1016/j.pscychresns.2008.07.010 PMC4153735 19394205

[25] Jackson PL Meltzoff AN Decety J How do we perceive the pain of others? A window into the neural processes involved in empathy. Neuroimage (2005) 24(3):771–9. 10.1016/j.neuroimage.2004.09.006 15652312

[26] Lamm C Decety J Singer T Meta-analytic evidence for common and distinct neural networks associated with directly experienced pain and empathy for pain. Neuroimage (2011) 54(3):2492–502. 10.1016/j.neuroimage.2010.10.014 20946964

[27] Cheng Y Lin CP Liu HL Hsu YY Lim KE Hung Expertise modulates the perception of pain in others. Curr Biol (2007) 17(19):1708–13. 10.1016/j.cub.2007.09.020 17900903

[28] Decety J Yang CY Cheng Y Physicians down-regulate their pain empathy response: an event-related brain potential study. Neuroimage (2010) 50(4):1676–82. 10.1016/j.neuroimage.2010.01.025 20080194

[29] Tomova L Majdandžić J Hummer A Windischberger C Heinrichs M Lamm C Increased neural responses to empathy for pain might explain how acute stress increases prosociality. Soc Cogn Affect Neurosci (2017) 12(3):401–8. 10.1093/scan/nsw146 PMC5465825 27798249

[30] Enzi B Amirie S Brüne M Empathy for pain-related dorsolateral prefrontal activity is modulated by angry face perception. Exp Brain Res (2016) 234(11):3335–45. 10.1007/s00221-016-4731-4 27447790

[31] Dinsdale N Crespi BJ The borderline empathy paradox: evidence and conceptual models for empathic enhancements in borderline personality disorder. J Pers Disord (2013) 27(2):172–95. 10.1521/pedi_2012_26_071 23514182

[32] Bohus M Limberger M Ebner U Glocker FX Schwarz B Wernz M Pain perception during self-reported distress and calmness in patients with borderline personality disorder and self-mutilating behavior. Psychiatry Res (2000) 95(3):251–60. 10.1016/S0165-1781(00)00179-7 10974364

[33] Schmahl C Greffrath W Baumgärtner U Schlereth T Magerl W Philipsen A Differential nociceptive deficits in patients with borderline personality disorder and self-injurious behavior: laser-evoked potentials, spatial discrimination of noxious stimuli, and pain ratings. Pain (2004) 110(1):470–9. 10.1016/j.pain.2004.04.035 15275800

[34] Ludäscher P Bohus M Lieb K Philipsen A Jochims A Schmahl C Elevated pain thresholds correlate with dissociation and aversive arousal in patients with borderline personality disorder. Psychiatry Res (2007) 149(1):291–6. 10.1016/j.psychres.2005.04.009 17126914

[35] van der Heiden L Scherpiet S Konicar L Birbaumer N Veit R Inter-individual differences in successful perspective taking during pain perception mediates emotional responsiveness in self and others: an fMRI study. Neuroimage (2013) 65:387–94. 10.1016/j.neuroimage.2012.10.003 23063451

[36] Schmahl C Bohus M Esposito F Treede RD Di Salle F Greffrath W Neural correlates of antinociception in borderline personality disorder. Arch Gen Psychiatry (2006) 63(6):659–66. 10.1001/archpsyc.63.6.659 16754839

[37] Wittchen HU Zaudig M Fydrich T Strukturiertes Klinisches Interview für DSM-IV. Göttingen: Hogrefe Verlag (1997).

[38] Lehrl S Merz J Burkard G Fischer B Manual zum MWT-a [Manual for MWT-a]. Erlangen, Germany: Perimed (1991).

[39] Baddeley A Emslie H Nimmo-Smith I The Spot-The-Word Test: a robust estimate of verbal intelligence on lexical decision. Br J Clin Psychol (1993) 32:55–65. 10.1111/j.2044-8260.1993.tb01027.x 8467274

[40] Lamm C Nusbaum HC Meltzoff AN Decety J What are you feeling? Using functional magnetic resonance imaging to assess the modulation of sensory and affective responses during empathy for pain. PLoS One (2007) 2(12):e1292. 10.1371/journal.pone.0001292 18091986 PMC2144768

[41] Friston KJ Holmes AP Worsley KJ Poline JP Frith CD Frackowiak RS Statistical parametric maps in functional imaging: a general linear approach. Hum Brain Mapp (1994) 2(4):189–210. 10.1002/hbm.460020402

[42] Friston KJ Fletcher P Josephs O Holmes A Rugg MD Turner R Event-related fMRI: characterizing differential responses. Neuroimage (1998) 7(1):30–40. 10.1006/nimg.1997.0306 9500830

[43] Poldrack RA Baker CI Durnez J Gorgolewski KJ Matthews PM Munafò MR Scanning the horizon: towards transparent and reproducible neuroimaging research. Nat Rev Neurosci (2017) 18(2):115–26. 10.1038/nrn.2016.167 PMC6910649 28053326

[44] Lissek S Peters S Fuchs N Witthaus H Nicolas V Tegenthoff M Cooperation and deception recruit different subsets of the theory-of-mind network. PloS One (2008) 3(4):e2023. 10.1371/journal.pone.0002023 18431500 PMC2295259

[45] Maldjian JA Laurienti PJ Kraft RA Burdette JH An automated method for neuroanatomic and cytoarchitectonic atlas-based interrogation of fMRI data sets. Neuroimage (2003) 19(3):1233–9. 10.1016/S1053-8119(03)00169-1 12880848

[46] Tzourio-Mazoyer N Landeau B Papathanassiou D Crivello F Etard O Delcroix Automated anatomical labeling of activations in SPM using a macroscopic anatomical parcellation of the MNI MRI single-subject brain. Neuroimage (2002) 15(1):273–89. 10.1006/nimg.2001.0978 11771995

[47] Brett M Anton JL Valabregue R Poline JB Region of interest analysis using an SPM toolbox. In: 8th international conference on functional mapping of the human brain. vol. 16 Sendai, Japan (2002). p. 497.

[48] Kriegeskorte N Simmons WK Bellgowan PS Baker CI Circular analysis in systems neuroscience: the dangers of double dipping. Nat Neurosci (2009) 12(5):535. 10.1038/nn.2303 19396166 PMC2841687

[49] Costantini M Galati G Romani GL Aglioti SM Empathic neural reactivity to noxious stimuli delivered to body parts and non-corporeal objects. Eur J Neurosci (2008) 28(6):1222–30. 10.1111/j.1460-9568.2008.06406.x 18783380

[50] Phillips ML Williams LM Heining M Herba CM Russell T Andrew C Differential neural responses to overt and covert presentations of facial expressions of fear and disgust. Neuroimage (2004) 21(4):1484–96. 10.1016/j.neuroimage.2003.12.013 15050573

[51] Fusar-Poli P Placentino A Carletti F Landi P Allen P Surguladze S Functional atlas of emotional faces processing: a voxel-based meta-analysis of 105 functional magnetic resonance imaging studies. J Psychiatry Neurosci (2009) 34(6):418 https://www.ncbi.nlm.nih.gov/pubmed/?term=Fusar-Poli++Functional+atlas+of+emotional+faces+processing%3A+a+voxel-based+​meta-analysis 19949718 PMC2783433

[52] Vytal K Hamann S Neuroimaging support for discrete neural correlates of basic emotions: a voxel-based meta-analysis. J Cogn Neurosci (2010) 22(12):2864–85. 10.1162/jocn.2009.21366 19929758

[53] Niedtfeld I Schulze L Kirsch P Herpertz SC Bohus M Schmahl C Affect regulation and pain in borderline personality disorder: a possible link to the understanding of self-injury. Biol Psychiatry (2010) 68(4):383–91. 10.1016/j.biopsych.2010.04.015 20537612

[54] Schulze L Domes G Krüger A Berger C Fleischer M Prehn K Neuronal correlates of cognitive reappraisal in borderline patients with affective instability. Biol Psychiatry (2011) 69(6):564–73. 10.1016/j.biopsych.2010.10.025 21195392

[55] Ruocco AC Amirthavasagam S Choi-Kain LW McMain SF Neural correlates of negative emotionality in borderline personality disorder: an activation-likelihood-estimation meta-analysis. Biol Psychiatry (2013) 73(2):153–60. 10.1016/j.biopsych.2012.07.014 22906520

[56] Brüne M Borderline Personality DisorderWhy ‘fast and furious’? Evol Med Public Health (2016) 1:52–66. 10.1093/emph/eow002 PMC4782519 26929090

