# Supplementary material for: Enhanced Processing of Painful Emotions in Patients With Borderline Personality Disorder: A Functional Magnetic Resonance Imaging Study
Source: Front Psychiatry. 2019 May 27;10:357. doi: 10.3389/fpsyt.2019.00357 (PMC6545793; doi:10.3389/fpsyt.2019.00357)

**Supplemental material**

Table S1. Dependent *t*test for emotion*group interactions using emotions collapsed over pain and non-painful conditions for ROIs determined by the contrast [positive effect of pain]. Results for comparisons within the two groups separated are shown.

| **Group** | **ROI** | **Comparison Emotions** | ***t*** | ***df*** | ***p*** |
| --- | --- | --- | --- | --- | --- |
| BPD | Right supramarginal gyrus | Angry vs. painful | -2.38 | 19 | 0.028 |
|  |  | Happy vs. no emotion | 2.14 | 19 | 0.045 |
|  |  | Painful vs. no emotion | 2.54 | 19 | 0.020 |
|  | Left supramarginal gyrus | Angry vs. happy | -3.13 | 19 | 0.004 |
|  |  | Happy vs. neutral | 2.98 | 19 | 0.008 |
|  |  | Happy vs. no emotion | 4.17 | 19 | 0.001 |
|  |  | Painful vs. no emotion | 2.31 | 19 | 0.032 |
|  |  | Neutral vs. no emotion | 3.17 | 19 | 0.005 |
| HC | Right supramarginal gyrus | Angry vs. painful | 2.31 | 18 | 0.033 |
|  | Left supramarginal gyrus | Angry vs. happy | 2.26 | 18 | 0.037 |
|  |  | Angry vs. painful | 3.19 | 18 | 0.005 |
|  |  | Painful vs. neutral | -2.65 | 18 | 0.016 |

Table S2. Dependent *t*test for emotions between painful and non-painful conditions within the BPD and HC groups. Results are shown for ROIs determined by the contrast [positive effect of pain].

| **Group** | **ROI** | **Comparison Emotions** | ***t*** | ***df*** | ***p*** |
| --- | --- | --- | --- | --- | --- |
| BPD | Left insula | neutral face+pain vs. neutral face+nopain | 3.30 | 19 | 0.004 |
|  | Right supramarginal gyrus | neutral face+pain vs. neutral face+nopain | 2.29 | 19 | 0.034 |
| HC | Right supramarginal gyrus | no emotion+pain vs. no emotion+ nopain | 3.35 | 18 | 0.004 |

Table S3. Results of mixed model ANOVAs with the factors pain condition (pain/no pain), emotion (angry, happy, neutral, and painful, no emotion) and medication (BPD with medication/BPD free of medication), for each region separately. The Table shows all significant main effect and interactions found by this analyses. Non-significant results are not shown.

| **ROI** | **main effect** | **Interaction** | ***F*** | ***df*** | ***p*** |
| --- | --- | --- | --- | --- | --- |
| Left Insula |  | condition* emotion | 4.57 | 2.13 | 0.015 |
| Left thalamus |  |  |  |  |  |
| Right supramarginal gyrus | condition |  | 6.67 | 1, 18 | 0.019 |
|  | emotion |  | 4.49 | 2.13, 38.30 | 0.016 |
|  |  | emotion*medication | 5.90 | 2.13 | 0.005 |
| Left supramarginal gyrus | condition |  | 20.30 | 1, 18 | < 0.001 |
|  | emotion |  | 6.09 | 2.28, 49.96 | 0.002 |
|  |  | condition*emotion | 2.11, 37.90 | 4.52 | 0.016 |

For additional analysis, we entered all facial expressions followed by pain/no pain in an ANOVA model in SPM and calculated the interaction contrast [group*emotion]. Each condition encompasses 24 trials, therefore the statistical threshold was set to *p*[uncorr.] < 0.005 for visualization. Only activations surviving a threshold of *p*[uncorr.] < 0.001 were considered as relevant. We discovered activations in the supramarginal gyrus bilaterally and the left anterior insula. In addition, we extracted the signal change expressed in Arbitrary Units (A.U.) using SPM. : For the left anterior insula, the size of the activated region was 8 voxels with *F* = 5.58, *Z* = 3.10, *p*_(uncorr)_ = 0.001 (peak-level). The activation of the left supramarginal gyrus comprised 27 voxels with *F* = 5.78, *Z* = 3.17, *p*_(uncorr)_ = 0.001 and the right supramarginal gyrus contained 40 voxels with *F* = 6.30, *Z* = 3.37, *p*_(uncorr)_ = 0.000 (see Figure S1).

Figure S1: Activation and signal change in A.U. for regions derived from the contrast [interaction group X Emotion] for patients with BPD and healthy controls (HC). Regions shown are the left anterior insula (a, b) and the left and right supramarginal gyri (c-f). The diagram in b) and d) show differences between groups for pain and no pain conditions with preceding facial emotions and e) and f) show differences between groups for the facial expressions in the left and right supramarginal gyrus independent of pain condition.

Error bars represent SEM.


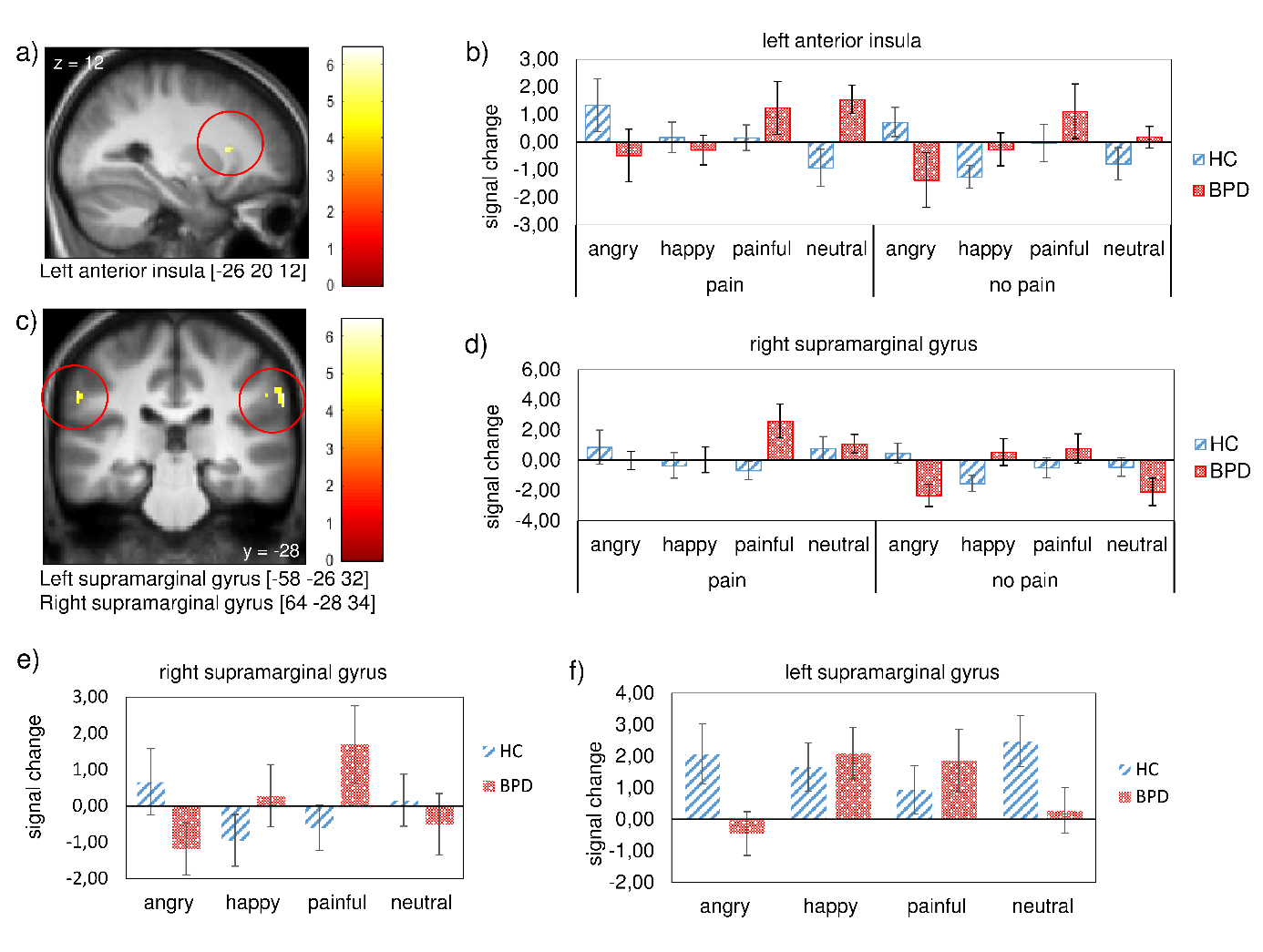


In order to check the reliability of the analysis, we repeated the statistical analyses with data extracted from anatomical ROI for the left anterior insula, the left supramarginal gyrus and the right supramarginal gyrus. The anatomical ROI were constructed using the WFU PickAtlas toolbox and based on the AAL atlas. The results of mixed model ANOVAs are shown in Table S4 and Figure S2.

Table S4. Results of mixed model ANOVAs with the factors pain condition (pain/no pain), emotion (angry, happy, neutral, and painful, no emotion) and group (BPD/HC), for each region separately. The Table shows all significant main effect and interactions (Greenhouse-Geisser corrected) found by this analyses. Non-significant results are not shown.

| **ROI** | **main effect** | **Interaction** | ***F*** | ***df*** | ***p*** |
| --- | --- | --- | --- | --- | --- |
| Right supramarginal gyrus | condition |  | 6.23 | 1, 37 | 0.017 |
|  |  | emotion*group | 4.66 | 2.78 | 0.005 |
|  |  | condition*emotion*group | 3.01 | 3.49 | 0.026 |
| Left supramarginal gyrus | condition |  | 23.98 | 1, 37 | > 0.001 |
|  |  | emotion*group | 3.38 | 2.92 | 0.022 |
|  |  | condition*emotion*group | 3.52 | 2.81 | 0.034 |

Figure S2: Signal change in % for patients with BPD and healthy controls (HC). Regions shown are the left supramarginal gyrus (a) and the right supramarginal gyrus (b). The diagrams on the left show differences between groups for pain and nopain conditions with preceding facial emotions and diagrams on the right show differences between groups for the facial expressions in the left and right supramarginal gyrus independent of pain condition. Significant differences between pain conditions for emotions are not marked.

Error bars represent SEM and * *p* < 0.05, ** *p* <0.010, *** *p* < 0.001.


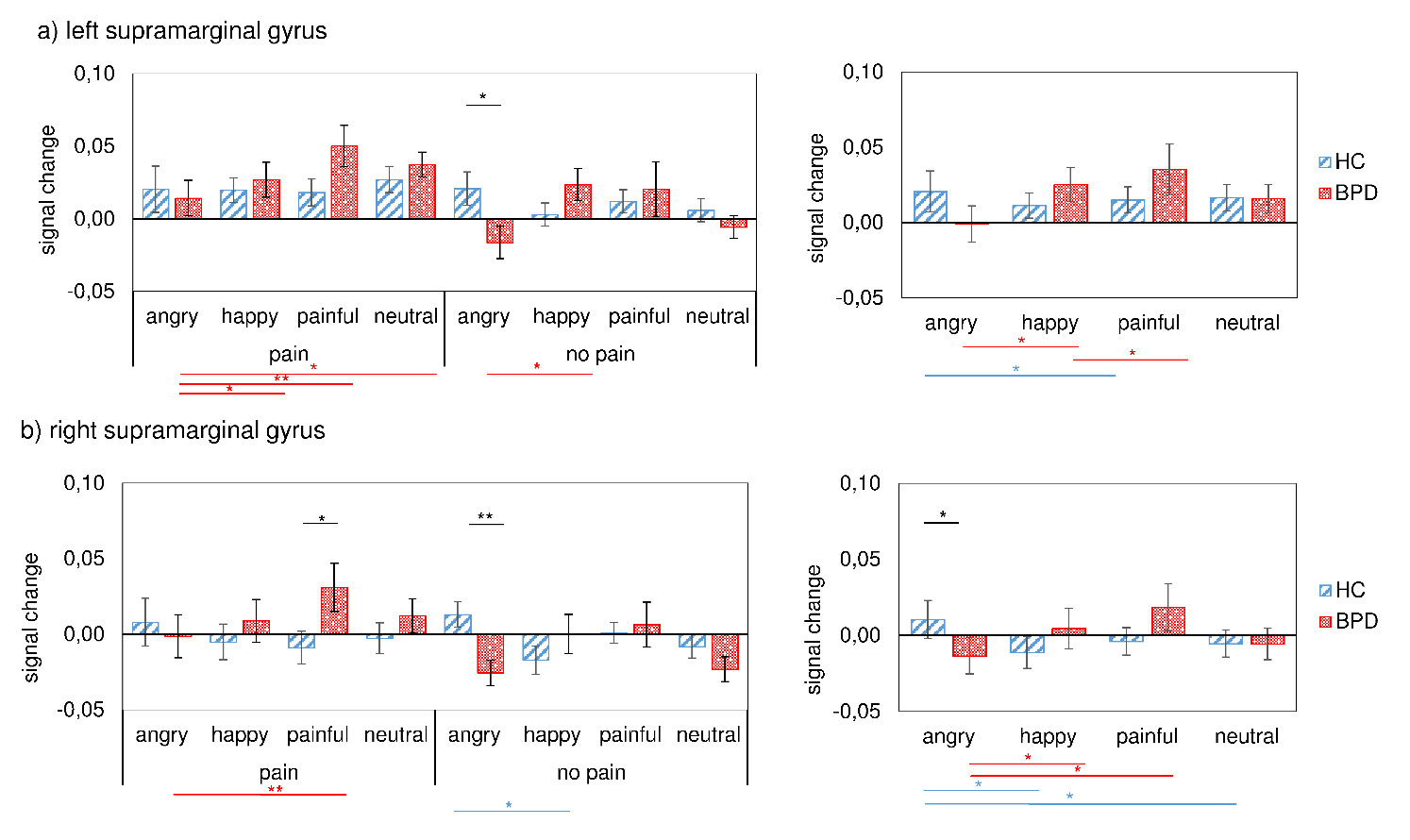

Supplement: Supplementary file 1 [file DataSheet_1.docx]
